# Genotify: Fast, lightweight gene lookup and summarization

**DOI:** 10.21105/joss.00885

**Published:** 2018-08-15

**Authors:** Jared M. Andrews, Mohamed El-Alawi, Jacqueline E. Payton

**Affiliations:** 1 Washington University School of Medicine, Department of Pathology and Immunology; 2 None

## Motivation

With the advent of low-cost, massively parallel sequencing, researchers are often faced with the task of manually curating lists of significant genes to find points of biological interest for further study. Determining the protein product function and biological significance in the context of the study for these genes can consume significant time and effort. Despite dozens of data sources that provide gene annotations, including genomic mapping, aliases, expression, function, disease associations, ontology terms, and more, accessing this information requires combing through these databases as well as the knowledge of their existence. While many databases provide APIs for high-throughput annotation (e.g. UniProtKB (Bateman et al., 2017), NCBI Gene (Brown et al., 2015), and Ensembl (Zerbino et al., 2018)), there exist few non-programmatic options for querying and collating information from multiple databases for everyday use. Genotify addresses this unmet need, providing an intuitive GUI with flexible search options that intelligently queries both general and species-specific databases to expedite manual curation and enable convenient routine gene lookup.

## Summary

**Figure 1:**
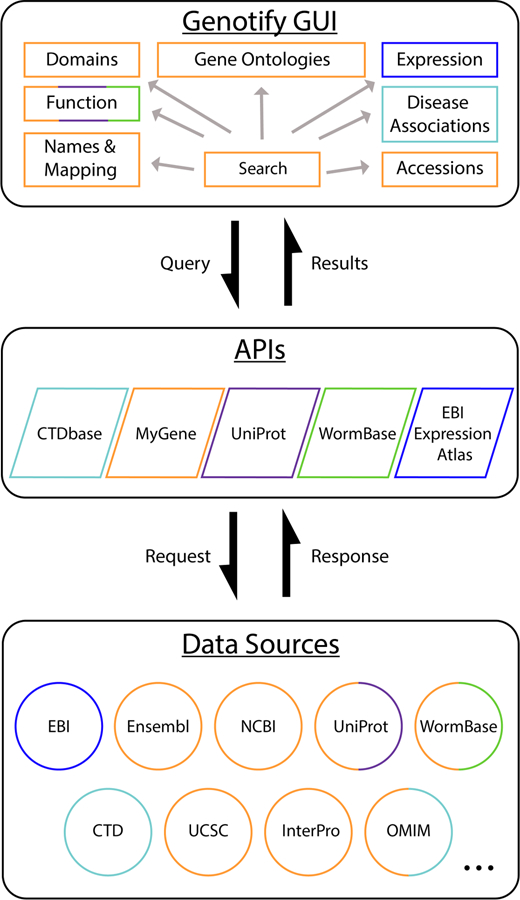
Schematic showing design and workflow of Genotify. Colors indicate connections between results fields, APIs, and data sources. API queries and data sources are determined dynamically based on availability and species.

Genotify is a lightweight desktop application that provides rapid gene lookup and summarized annotations from dozens of major and specialized data sources (Figure 1). Initial gene queries are submitted to the MyGene.info API, which collects gene annotation data from over 30 data sources (Xin et al., 2016). Additional API calls are made depending on species and the accessions returned by the MyGene.info API. Gene symbols, names, chromosomal coordinates or IDs (Entrez, Ensembl, etc) are all viable query terms, and the results returned in JSON format are quickly parsed and displayed to the user. Results are sortable, searchable, and navigable with a single click. Genotify supports queries for all species, though the information available for each differs significantly. The UniProtKB API is used to collect additional functional information from the curated Swiss-Prot database when available (Bateman et al., 2017). The EBI Expression Atlas widget dis- plays interactive expression data for several species (Petryszak et al., 2016), and the ProtVista protein viewer provides a wealth of interactive protein data including domains, post-translation modifications, and impact of known genetic variants (Watkins, Garcia, Pundir, Martin, & Valencia, 2017). Disease associations for human genes are collected with the Comparative Toxicogenomics Database (CTDbase) API (A. P. Davis et al., 2017). Organism-specific databases like WormBase and CTDbase are utilized when appropriate (Lee et al., 2018). Importantly, directly querying major databases ensures that the information Genotify returns is always up to date and removes the need for manual updates of locally stored flat database files.

Genotify is a GUI desktop application built on the Electron Javascript framework, which allows for inherent cross-platform deployment to 32 or 64-bit linux, OSX, and Windows systems. Genotify’s use of existing APIs means no data sources are downloaded, saving disk space and making installation simple. Users can limit their search to one or many species, and a hotkey command can directly query terms from the clipboard for ease of use.

## Use cases

We designed Genotify for experimentalists and bioinformaticists who need an up-to-date, comprehensive summary of a gene’s annotation, function, expression, ontology, and dis- ease associations in a single location. Our group uses it daily to facilitate:

- rapid, efficient lookup of genes while reviewing literature or curating lists of significant genes,
- close investigation of families of related genes,
- quick ascertainment of the biological significance of differentially expressed genes or associating proteins,
- determination of known disease associations,
- exploration of protein structure, modifications, and variants,
- comparison of mRNA expression of a queried gene across diverse tissues, cell types, and species.

## Availability

Genotify is released under the GPL-3.0 license with source code and binaries freely available at https://github.com/j-andrews7/Genotify, implemented as a desktop application built on the Electron framework and supported on linux, OS X, and MS Windows.
